# Cyclopedia of Obstetrics and Gynecology

**Published:** 1888-08

**Authors:** 


### Book Reviews.

CYCLOPEDIA OF OBSTETRICS AND GYNÆCOLOGY. Vols. V. to XII. inclusive. Edited by Egbert H. Grandin, M. D., Obstetric Surgeon to the New York Maternity Hospital, etc. New York: Wm. Wood & Co. 1887.

The first four volumes of this work, relating to obstetrics, have already been reviewed in this journal. The remaining eight volumes are upon the subject of gynæcology, and they cover the field fully and satisfactorily. Volume V. contains a discussion of Gynæcological Diagnosis and General Gynæcological Therapeusis, by Chrobak, of Vienna, and a Treatise on Electricity in Gynæcology and Obstetrics, by Grandin. Volumes VI. and VII. consist of a translation of Hegar and Kaltenbach's valuable Handbook of General and Operative Gynæcology. Volume VIII. is by Olshausen, and treats of Diseases of the Ovaries. Volume IX. contains translations of Billroth on Diseases of the Female Mammary Glands, and Gusserow on New Growths of the Uterus. Volume X. contains Diseases of the Female Urethra and Bladder, by Winckel, and Diseases of the Vagina by Breisky. Volume XI., Sterility and Developmental Anomalies of the Uterus, by Muller, and the Menopaüse by Bonner. Volume XII. completes the series with a treatise by Bandl on Diseases of the Tubes, Ligaments, Pelvic Peritoneum and Pelvic Cellular Tissue and Extra-uterine Pregnancy, and by Zweifel on Diseases of the External Female Genitals and Lacerations of the Perineum.

Thus it will be seen that this series furnishes a library of standard works by the best authors on the subject of gynæcology. It is superior to any single work on diseases of women in the English language, since each article is a complete and exhaustive monograph in itself. The greatest fault to be found is that all the authors are German, and hence the work presents only the German aspect of the subject, which in some respects is widely different from the American. There is also some repetition and overlapping of subjects. But this perhaps is unavoidable in a collection of separate monographs.

Taken all in all, this is by far the most valuable library that Wm. Wood & Co. have issued. To the non-German-reading portion of the profession it opens the works of authors whose names have long been familiar, but whose writings heretofore have been inaccessible. Moreover, the book is free from the hasty, perfunctory, written-to-order style which has characterized a number of the "systems" recently published in this country. V. O. H.

